# Spatially rearranged object parts can facilitate perception of intact whole objects

**DOI:** 10.3389/fpsyg.2014.00482

**Published:** 2014-05-27

**Authors:** Laura Cacciamani, Alisabeth A. Ayars, Mary A. Peterson

**Affiliations:** ^1^Department of Psychology, University of ArizonaTucson, AZ, USA; ^2^Cognitive Science Program, University of ArizonaTucson, AZ, USA

**Keywords:** figure-ground perception, part-whole processing, spatially rearranged parts, object perception, masked priming

## Abstract

The familiarity of an object depends on the spatial arrangement of its parts; when the parts are spatially rearranged, they form a novel, unrecognizable configuration. Yet the same collection of parts comprises both the familiar and novel configuration. Is it possible that the collection of familiar parts activates a representation of the intact familiar configuration even when they are spatially rearranged? We presented novel configurations as primes before test displays that assayed effects on figure-ground perception from memories of intact familiar objects. In our test displays, two equal-area regions shared a central border; one region depicted a portion of a familiar object. Previous research with such displays has shown that participants are more likely to perceive the region depicting a familiar object as the figure and the abutting region as its ground when the familiar object is depicted in its upright orientation rather than upside down. The novel primes comprised either the same or a different collection of parts as the familiar object in the test display (part-rearranged and control primes, respectively). We found that participants were more likely to perceive the familiar region as figure in upright vs. inverted displays following part-rearranged primes but not control primes. Thus, priming with a novel configuration comprising the same familiar parts as the upcoming figure-ground display facilitated orientation-dependent effects of object memories on figure assignment. Similar results were obtained when the spatially rearranged collection of parts was suggested on the groundside of the prime's border, suggesting that familiar parts in novel configurations access the representation of their corresponding intact whole object before figure assignment. These data demonstrate that familiar parts access memories of familiar objects even when they are arranged in a novel configuration.

## Introduction

Objects consist of collections of parts, but objects are also usually encountered in a particular orientation and with their parts arranged in a particular way. For instance, when we perceive or identify a table lamp, it is usually the case that the base of the lamp is on the table and is topped with the lampshade. The visual system may capitalize on the regularity of the spatial arrangement of object parts by representing objects not only (or not at all) by their individual parts, but also (or solely) by the spatial arrangement of those parts (Biederman, 1987; Hummel and Biederman, 1992).

An interesting question is whether object parts must be arranged in their proper configuration in order for the familiar object to be correctly identified. Prior research suggests that indeed, the spatial arrangement of object parts matters for object identification (Hoffman and Richards, 1984; Biederman, 1987; Hummel and Biederman, 1992; Saiki and Hummel, 1998; Arguin and Saumier, 2004) and also for face perception (Liu et al., 2010). For instance, when the parts of an object are spatially rearranged into a novel configuration, participants are slower to identify the object than when the parts are in their proper configuration (Cave and Kosslyn, 1993), suggesting that object identification cannot merely be a matter of recognizing the collection of parts of which the object is composed. Consistent with this, research on face recognition shows that faces are perceived as wholes rather than a collection of individual parts (Tanaka and Farah, 1993; Farah et al., 1998). Moreover, Poljac et al. (2012) (Suchow and Alvarez, 2011) found that awareness of object parts is reduced when a Gestalt image (a perceptual whole) emerges from the parts, again suggesting that explicit object perception is independent of part perception, at least under some conditions and perhaps depending on the relationship between a part and the whole. According to Pomerantz (1981), two types of part-whole relationships exist: in Type P relationships, only the *position* of the parts matters for the whole configuration, whereas in Type N relationships, the position but also the *nature* of the parts is important for identifying the whole. Poljac et al.'s object parts were small, colored dots that either formed a human figure or a random display (cf., Suchow and Alvarez, 2011). Thus, only the position (not the nature) of the dots informed the whole—these were Type P relationships. In the present study, we are interested in Type N part-whole relationships in which both the position and the nature of the parts matter for the whole.

The separation of object part representations from representations of object wholes is also supported by work showing that individual parts and their spatial arrangement are differentially represented in the brain. For example, single-cell recording studies in monkeys have shown that neurons in the inferior temporal (IT) cortex respond selectively either to individual object parts (i.e., invariant of configuration) or the configuration they form (i.e., invariant of part identity), but not both (Baker et al., 2002; Yamane et al., 2006). Additional evidence of differential neural representations for parts and configurations comes from neuropsychological studies on different patient populations. Patients with integrative agnosia, for instance, are able to perceive object parts but are impaired at assembling those parts into a meaningful, recognizable whole. As a result, integrative agnosics will rely on local parts to identify objects; in this way, they might identify a harmonica as a computer based on the “keys” present in both objects (Riddoch and Humphreys, 1987; Behrmann and Kimchi, 2003). The opposite deficit also exists—that is, impairment of part but not whole recognition. For example, in a condition known as simultanagnosia, processing is limited to one item at a time (Luria, 1959); however, the definition of “item,” in this case, is not restricted to full objects. That is, patients with simultanagnosia have been shown to rely on global properties in recognizing objects; in this way, if object recognition requires assessment of the local parts, simultanagnosics are impaired (Riddoch and Humphreys, 2004). It has been posited that these recognition deficits reflect the differential systems involved in processing whole objects vs. their individual parts, with integrative agnosics impaired on the former (Behrmann et al., 2006) and simultanagnosics impaired on the latter (Riddoch and Humphreys, 2004). These studies reveal that parts and wholes have separable representations in the brain, and although these representations likely interact, the familiarity of whole configurations ultimately underlies explicit object recognition and perception in neurologically intact individuals. In this way, if the parts of a familiar object are spatially rearranged, the resulting configuration is perceived as novel.

These previous studies suggest that explicit identification of whole objects depends on the spatial arrangement of the parts, but new evidence suggests that parts of an object—even when in a novel configuration—may nevertheless activate a representation of the whole object that is not available to conscious awareness but still factors in object perception. This research has shown that medial temporal lobe (MTL) structures—specifically, the perirhinal cortex (PRC) and anterior temporal lobe (ATL)—assess the familiarity of both parts and configurations (Barense et al., 2012; Peterson et al., 2012a). Using functional magnetic resonance imaging (fMRI), Peterson et al. (2012a) found that these MTL structures are differentially active to intact familiar configurations, in which the parts of a familiar object are present in their typical (familiar) spatial arrangement; novel configurations created by spatially rearranging the same familiar parts into a novel configuration; and novel configurations composed of novel parts. The differential activation of the PRC to novel configurations composed of novel vs. familiar parts indicates that the PRC is not merely sensitive to the familiarity or novelty of the whole configuration, but it also is sensitive to the familiarity or novelty of the parts constituting the configuration. Furthermore, Peterson et al.'s (2012a) results do not suggest that the PRC is sensitive to a continuum of familiarity (that the familiarity of both the parts and the whole configuration feed into), because familiarity had a non-monotonic relationship to PRC activation: Specifically, novel configurations composed of novel parts, although lowest in familiarity, elicited a magnitude of PRC activation that fell in between the magnitude elicited by familiar vs. novel configurations composed of familiar parts. These data suggest that the PRC receives and processes input from low-level areas where part familiarity is represented, in addition to input from levels where configurations are represented. However, it is not known whether these part and whole representations in the PRC are separate, or whether they interact. If these representations do interact, an intriguing question is whether the familiar parts of an object when rearranged to form a novel configuration can prime the representation of the whole configuration in which they are typically perceived. In the current study, we directly addressed this possibility by assessing whether a prior, brief presentation of a novel prime created by spatially rearranging the parts of a familiar object facilitates access to the representation of the whole familiar configuration typically formed by those parts.

**Figure 1:**
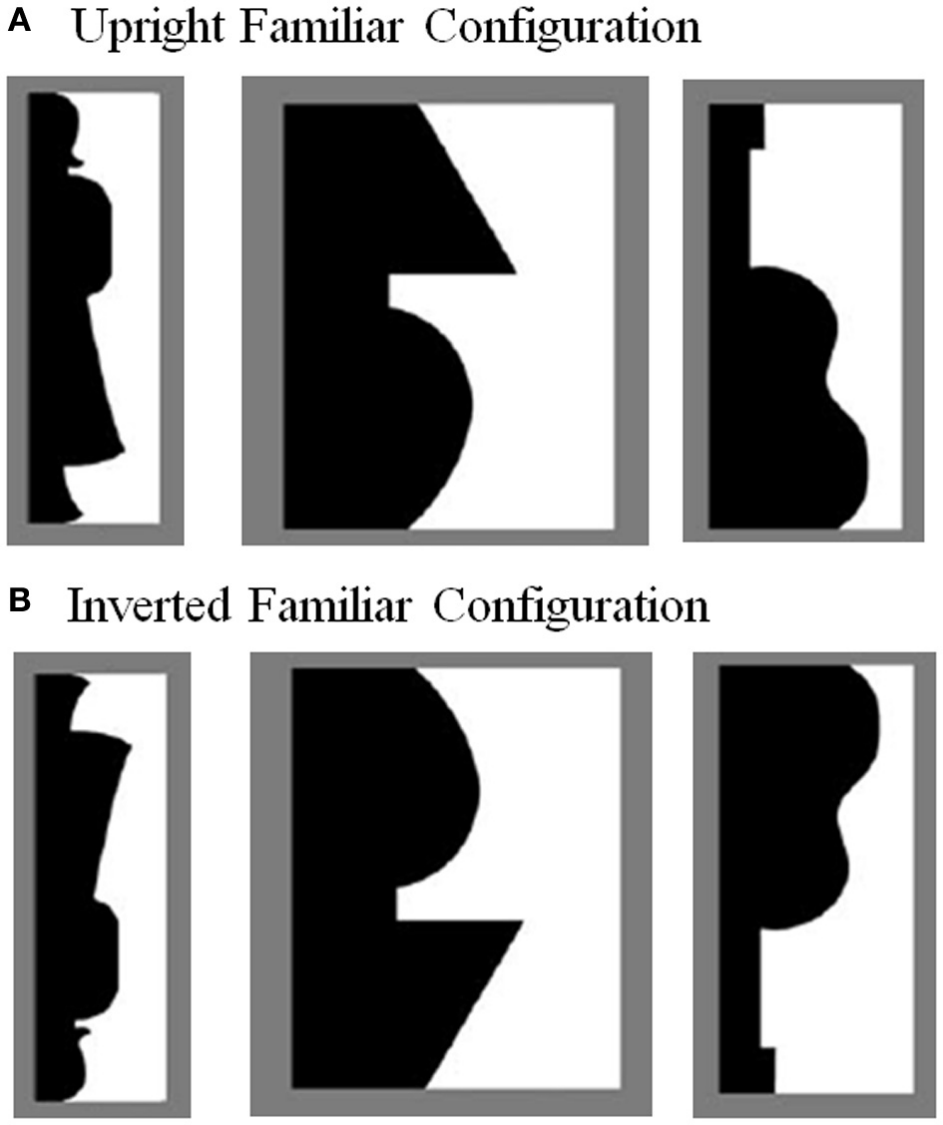
**Bipartite figure-ground test stimuli used in this study (see also Peterson et al., 1991, 1998, 2000; Gibson and Peterson, 1994; Barense et al., 2012)**. Familiar configurations were either **(A)** upright or **(B)** inverted. For display purposes, the familiar regions here are always shown in black and on the left (this was fully counterbalanced in the experiment). Shown here in the critical regions are, from left to right, a standing woman, a lamp, and a guitar. Figure modified and reproduced with permission from Gibson and Peterson (1994), Peterson et al. (1998, 2000), Barense et al. (2012).

Although no studies have addressed the influence of object parts on perception of the whole in this manner, previous research has found that representations of familiar objects can indeed be accessed implicitly and can influence perception (Peterson et al., 1991, 1998, 2000; Gibson and Peterson, 1994; Peterson and Gibson, 1994a,b; Barense et al., 2012). To assess implicit access to and facilitation of familiar configurations, these studies have used a figure assignment task. In this task, participants are shown bipartite displays (like those depicted in Figure 1) in which two equal-area regions share a central border. In this type of display, one region is typically perceived as the *figure* (or the object) shaped by the shared border, while the other region is perceived as a shapeless *ground* continuing behind that figure at their shared border. On each trial, participants are asked to report which region (left or right) they perceive as the figure. Using this paradigm, studies have shown that *figure assignment* is influenced by the presence of a portion of an intact familiar object on one side of the border. Specifically, the region that depicts a familiar object at the central border is more likely to be perceived as the figure at that border than a region that depicts a novel, meaningless shape (i.e., the left vs. right side of the border in the examples in Figure 1A). To ascertain that these effects were due to object memories *per se* rather than low-level stimulus features, these studies typically included a control condition in which the familiar configuration was inverted (i.e., rotated 180° from its canonical, upright orientation; see Figure 1B). Although object memories are still accessed for inverted familiar stimuli, this access is weak and takes time to accumulate, especially compared to object memory access for upright familiar stimuli (Jolicoeur, 1985; Corballis, 1988; Oram and Perrett, 1992). Because of this, when upright and inverted displays are used, the typical finding is that the figure is perceived on the side of a border depicting a portion of a familiar object significantly more often when the displays are upright vs. inverted (i.e., Figures 1A vs. 1B). This holds true even if reports in the inverted condition are above chance due to weak access to object memories (e.g., Peterson and Gibson, 1994a; Barense et al., 2012). The orientation dependency of these results indicates that figure assignment can be influenced by quickly-accessed object memories, as the only difference between upright and inverted familiar objects is that upright objects are more quickly matched to memory representations than inverted objects (i.e., the orientation manipulation holds constant any low-level factors that distinguish between the two regions of the bipartite displays). Henceforth, we will hereby refer to these effects as orientation-dependent *object memory effects on figure assignment*, or orientation-dependent *OMEFA* effects.

Critically, orientation-dependent OMEFA effects are only observed when the parts of the familiar object depicted in the bipartite display are arranged in their typical spatial arrangement. When the familiar parts are spatially rearranged (thereby forming a novel configuration), reports of that region as figure are not significantly above chance and are equivalent for upright and inverted displays (Peterson et al., 1991, 1998; Gibson and Peterson, 1994; Peterson and Gibson, 1994a,b).

This indicates that the sole presence of familiar parts in the absence of a familiar whole is typically not sufficient to produce effects on figure assignment (although brain damage can change this; Barense et al., 2012). In this previous research, “parts” of familiar configurations were defined as lying between successive minima of curvature as determined from inside the region depicting the familiar configuration (e.g., the head, arms, skirt, and feet of the standing woman depicted in Figure 1A; see Biederman, 1987, and Hoffman and Richards, 1984, for similar definitions of object parts). In the present studies, we use the same definition of part, as did Barense et al. (2012) and Peterson et al. (2012a).

Although orientation-dependent OMEFA effects are typically not observed when a part-rearranged novel object is suggested on one side of the central border of bipartite displays, Peterson et al.'s (2012a) fMRI research suggests that the familiarity of the collection of parts is still represented at high levels of the visual system. Thus, spatially rearranged familiar parts could still influence perception by activating the representation of the whole familiar configuration, which would only affect behavior in situations where the whole is presented.

Importantly, these orientation-dependent OMEFA effects (or lack thereof, in the case of bipartite displays where a novel configuration composed of familiar parts is suggested on one side of the central border) occur implicitly; explicit, declarative knowledge pertaining to the familiar configurations in these bipartite displays is neither necessary nor sufficient for these effects. This has been demonstrated in prior studies where telling participants up-front about the relationship between upright familiar configurations and their inverted or their part-rearranged counterparts did not bias participants' figure reports regarding those regions, suggesting that explicit knowledge is not sufficient to produce OMEFA effects (Peterson et al., 1991; Peterson and Gibson, 1994a). Furthermore, Peterson et al. (2000) found that the figure reports of a brain-damaged patient (a visual agnosic) whose ability to identify objects was severely impaired nevertheless revealed orientation-dependent OMEFA effects. Thus, this figure assignment task is fundamentally a test of implicit access to object memories, making it very different from assessments of explicit identification ability.

Here, we used a masked priming paradigm to search for evidence that these implicit orientation-dependent OMEFA effects can be facilitated by prior presentation of a novel configuration created by spatially rearranging the parts of the familiar object. Such evidence, if found, would indicate that representations of familiar whole objects are accessed by their (familiar) parts even when those parts are arranged in a novel configuration. To test this, we employed a figure assignment task using the upright and inverted familiar configuration bipartite displays (see Figure 1). Before each bipartite display, a novel, symmetric, enclosed outline of a silhouette appeared, which served as a prime. There were two priming conditions: (1) the control condition, in which the silhouette outline depicted a meaningless shape composed of parts that were unrelated to the parts of the upcoming bipartite display (see Materials and Methods for our definition of an object part); and (2) the part-rearranged condition, in which the silhouette outline depicted a meaningless shape created by rearranging the familiar parts of the familiar configuration in the upcoming bipartite display. Pilot testing confirmed that both the control and part-rearranged outline silhouettes that served as our primes were indeed perceived as novel: Fewer than 22% of pilot participants agreed on the identity of the object depicted by the border. Moreover, participants did not identify the parts as parts of familiar objects in either the control outline silhouettes or the part-rearranged outline silhouettes. Thus, we can be reasonably confident that participants perceived both types of primes as depicting novel shapes and did not consciously perceive the familiarity of the constituent parts of the part-rearranged silhouettes (see Materials and Methods for a description of the post-experiment questioning used to clarify participants' perception of the prime). Therefore, any influence from the rearranged parts in the prime on the perception of the bipartite display is likely implicit.

Different observers participated in the control prime condition and the part-rearranged prime condition. We were interested in comparing reports of the familiar region as figure for upright vs. inverted displays as a function of whether or not the preceding silhouette comprised the same or different parts (part-rearranged vs. control conditions, respectively). We predicted that if the collection of parts comprising the part-rearranged silhouette, though rearranged, nevertheless activated memory representations pertaining to the whole intact familiar configuration, then we should see a larger difference in figure reports on upright vs. inverted trials following the part-rearranged silhouette prime than the control silhouette prime.

## Experiment 1

### Materials and methods

#### Participants

A total of 55 undergraduate students from the University of Arizona participated in this experiment in order to partially fulfill course requirements (29 in the part-rearranged condition, and 26 in the control condition). All participants provided informed consent prior to the experiment and reported normal or corrected-to-normal visual acuity. An additional three participants were removed from the analysis for failing to respond on more than 15% of trials during the experiment.

#### Stimuli and apparatus

Stimuli consisted of bipartite test displays and outline silhouettes presented on a medium gray background. The black and white bipartite test displays (*N* = 32) were those used previously (Peterson and Gibson, 1991; Gibson and Peterson, 1994; Peterson et al., 1998, 2000; Barense et al., 2012; see Figure 1). In all test displays, the two regions were equal in area. A portion of a familiar, real-world object (listed in Supplementary Material)^1^ was sketched on one side of the central border (henceforth, we use the term “familiar region” for brevity). The familiar region occurred equally often in black and white and on the left and right sides of the central border. For each participant, half of the test displays (*N* = 16) were presented in their upright orientation; the rest were inverted. Each participant saw a specific test display (depicting a particular familiar configuration) only once, in either the upright or the inverted orientation. The specific familiar configurations in each orientation (upright/inverted), color (black/white), and location (left/right) were counterbalanced across participants. The two regions of each display were equated for area and matched for convexity (Gibson and Peterson, 1994). The bipartite test displays subtended 5.6° of visual angle in height (H) and an average of 2.7° in width (W) and were presented centrally. A pattern mask (50% black, 50% white) of the same height and width followed each display.

**Figure 2:**
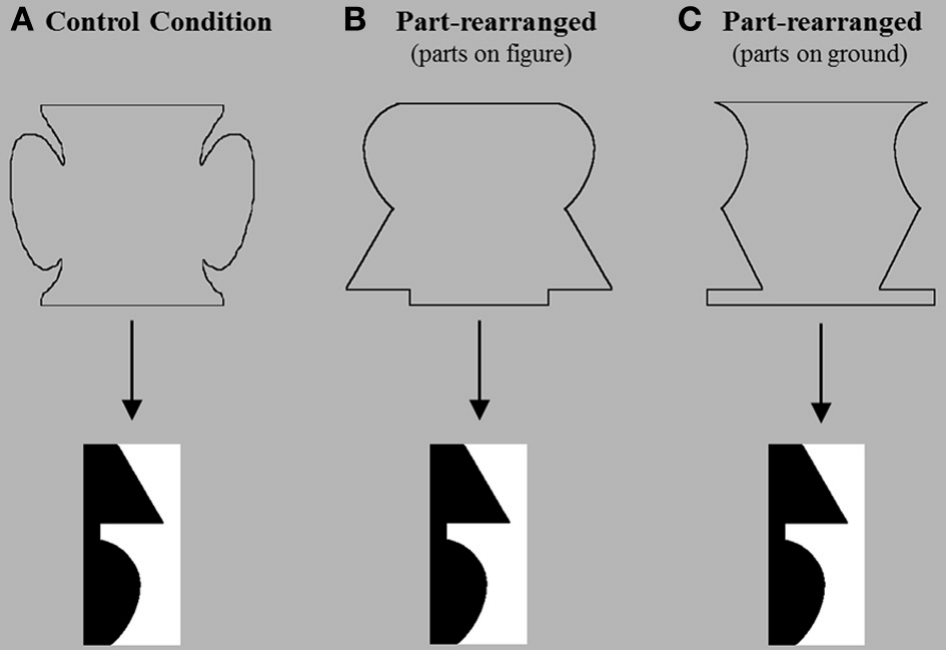
**Example stimuli in each priming condition**. In these examples, an upright lamp is depicted in the familiar region (here, in black and on the left side) of the bipartite display. Experiment 1 used **(A)** the control condition, in which the prime contains no parts of that lamp, and **(B)** the part-rearranged condition, in which the prime contains the lamp's familiar parts (i.e., the lampshade, the neck, and the base) rearranged into a novel configuration and depicted on the figure side of the vertical borders. Experiment 2 used **(C)** the part-rearranged condition in which the parts were suggested on the groundside of the outline prime's vertical borders.

The novel outline silhouette primes (*N* = 32) were small, enclosed, and symmetric—these attributes all favor perceiving the figure on the inside of the border. There were two types of primes: *control* primes and *part-rearranged* primes (Figures 2A,B, respectively). Both types of primes depicted novel, meaningless shapes on both the figure and ground sides of the silhouette border. For part-rearranged primes, the novel figure was created by spatially rearranging the parts of the familiar configuration in the upcoming bipartite display. The parts of these primes were found by identifying minima of curvature along the central border of the test displays as viewed from inside the region depicting the familiar object. Parts were defined as delimited by successive minima of curvature (cf., Hoffman and Richards, 1984; Biederman, 1987). We extracted these parts and rearranged them such that no two parts that were adjacent to one another in the intact familiar configuration were adjacent to one another in the part-rearranged novel configuration. This procedure produced a border that was a part-rearranged version of the border of the test displays. We then reflected the part-rearranged border around a vertical axis and drew a connecting line at the top and bottom to form an enclosed outline silhouette (see Figure 2B). For the control primes, the parts (and whole) depicted by the vertical borders were random novel shapes; no parts of the control prime (delimited by minima of curvature) were identical to any parts in the upcoming bipartite test display, although similar low-level features (i.e., angles and curvilinearity) may have been present. All prime silhouettes subtended 5.6° H × 6.8° W and were presented centrally. A pattern mask of the same size followed each prime.

A 21-in. Sony CRT monitor with a personal computer was used to present the stimuli and record responses. Participants viewed the monitor from a distance of 96 cm and utilized a chin rest to maintain their head position and viewing distance. Participants used a foot pedal to initiate each trial and to advance through the instructions. Responses were recorded using a custom response box with two horizontally arranged buttons. The presentation software was DMDX (Forster and Forster, 2003).

#### Design and procedure

Participants were assigned to either the part-rearranged silhouette prime condition or the control silhouette prime condition when they entered the lab (using an A-B-B-A order). Instructions were presented on the computer screen and were simultaneously read to the participant by the experimenter. Participants were told that on each trial, they would see an outline image appear briefly on the screen followed by a black and white display, and their task was to report whether they saw the figure lying on the right side or left side of the central border. The instructions defined the “figure” as the region of the display that looks like it has a definite shape and seems closer than the other region (the background) at the central border. Participants were told to respond using a button box with the left button labeled “left” and the right button labeled “right.”

**Figure 3:**
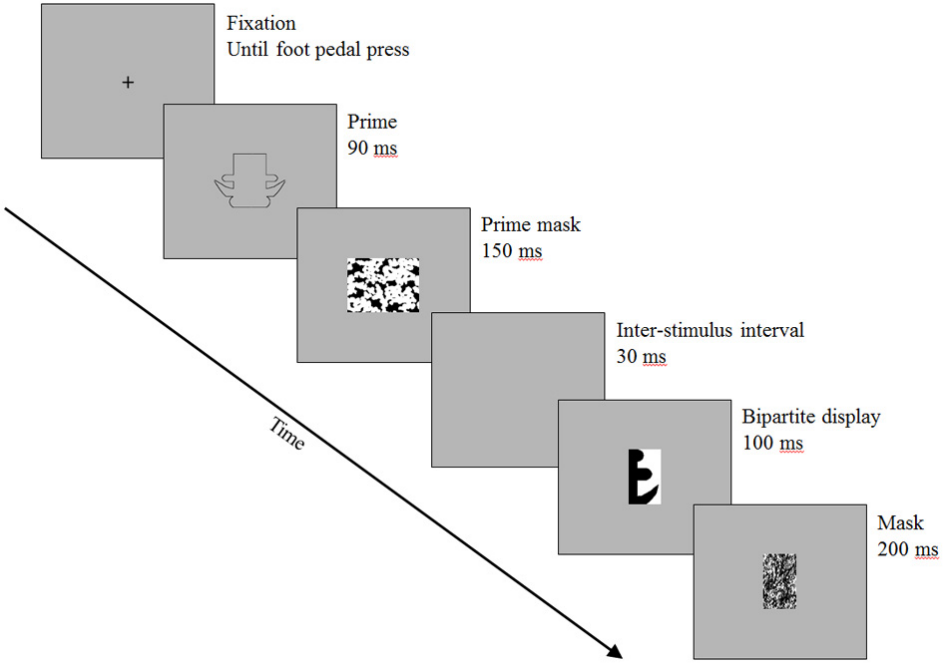
**Trial structure**. Shown here is an upright trial (an anchor is in black on the left side of the bipartite display) in the part-rearranged condition. Stimuli are enlarged with respect to the frame size for illustrative purposes.

A sample trial is shown in Figure 3. Each trial began with a black fixation cross that remained on the screen until the participant pressed the foot pedal. Upon foot pedal press, the outline silhouette prime (control or part-rearranged) was displayed for 90 ms, followed by a mask exposed for 150 ms. After an inter-stimulus interval of 30 ms, the bipartite display was presented for 100 ms and was followed by a 200 ms pattern mask. After participants made their figure response (or after 3000 ms elapsed), the fixation cross for the next trial appeared.

It should be noted that this trial structure differed from that of previous studies that have used these bipartite test displays (Gibson and Peterson, 1994; Peterson and Gibson, 1994a; Peterson et al., 2000; Barense et al., 2012). A number of these studies did not mask the bipartite test displays at all (Peterson et al., 2000; Barense et al., 2012). In the studies that did incorporate masking, a post-mask appeared after the bipartite test display, similar to the present experiment (Gibson and Peterson, 1994; Peterson and Gibson, 1994a); however, no previous studies included a pattern mask *before* the bipartite test display as well.

Our intention in including the mask after the outline silhouette was to abbreviate processing of the silhouette prime. However, the presence of the prime mask may have had other incidental effects. For example, although it was temporally separated from the bipartite display by a brief (30-ms) ISI, it could have served as a pre-mask for the bipartite test display and may have reduced the effective exposure duration of the display, rendering the figure assignment task more difficult than in previous studies. Indeed, our participants were asked in post-experiment questioning how easy or difficult the task was for them. The majority of participants reported having difficulty perceiving the bipartite test display due to the fast trial structure. This important point is one to which we will return in the Results section.

Before the experimental trials, participants were given 16 practice trials. No stimuli presented during the practice trials were used during the experimental trials. Thirty-two experimental trials followed the practice trials. The bipartite displays were upright for half of the trials (*N* = 16) and inverted for the remaining half (*N* = 16). Upright and inverted trials were randomly intermixed. Silhouette condition (control vs. part-rearranged) was manipulated between-subjects; thus, the type of silhouette remained constant across all 32 trials for a given participant.

After the experimental trials, participants were asked a series of post-experiment questions about their experience during the experiment. Specifically, they were shown an example of a prime (depending on what condition they were in) and were asked if they noticed it during the experiment. For participants in the part-rearranged prime condition, the experimenter pointed out and traced the familiar, rearranged parts in the sample outline prime and asked whether the participant ever noticed familiar parts in the outlines during the experiment. Participants were also asked whether they felt that the presence of the prime influenced their response to the black and white test display. Most participants reported that they barely noticed the prime due to its short exposure duration, the presence of the mask after the prime, and because their attention was focused on the bipartite test display and their demanding figure assignment task; moreover, they reported that they did not notice that the parts of the prime and the upcoming display were the same. Three participants in the part-rearranged condition indicated that they did perceive (or thought they might have perceived) the familiar parts contained in the primes, and that that the presence of those familiar parts might have influenced their decision on the figure-ground task. The data from these three participants were eliminated from the analysis, as the influence from the prime to the bipartite test display would have been explicit rather than implicit.

### Results and discussion

**Figure 4:**
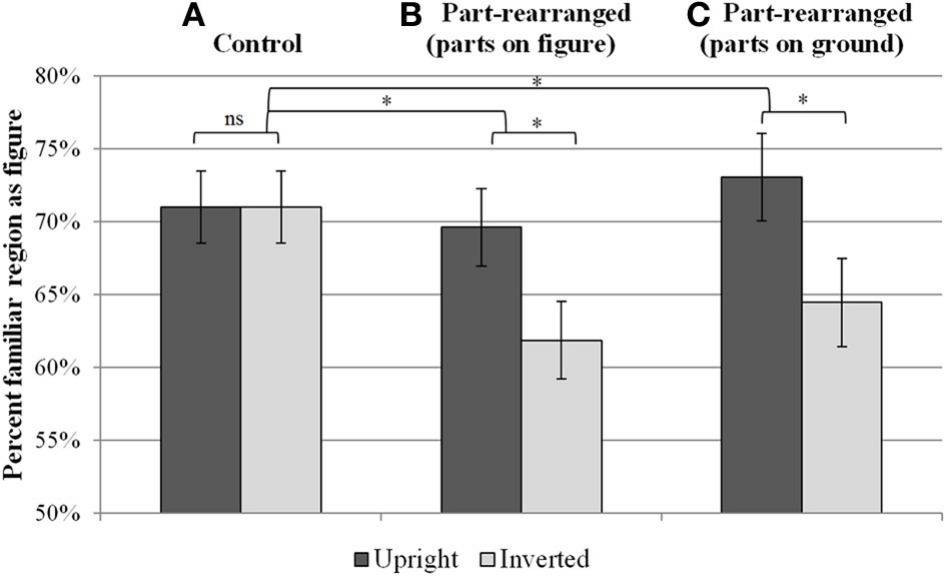
**Mean percent of trials on which the familiar region was reported as the figure for each of the between-subjects conditions**. Results of Experiment 1 are shown in **(A)** for the control condition and **(B)** for the part-rearranged condition in which the familiar parts were depicted on the figure side of the prime's borders. Results of Experiment 2 are shown in **(C)** for the part-rearranged condition in which the familiar parts were suggested in on the groundside of the prime's borders. Error bars represent standard error of the mean of the difference scores (upright-inverted). ^*^p < 0.05.

Figures 4A,B graphs the percentage of trials on which the familiar region was reported as the figure for each condition. Recall that our measure of interest was whether figure responses differed for upright and inverted displays, as this orientation effect is an index of OMEFA effects. We expected that the difference between upright and inverted would be greater following part-rearranged silhouette primes than control silhouette primes, because the former would activate the upright whole and would therefore lead to greater differential activation of the representation of the upright vs. inverted whole in that condition than in the control condition.

The results showed orientation-dependent OMEFA effects when bipartite displays were preceded by a part-rearranged outline silhouette (Figure 4B) but not when they were preceded by a control outline silhouette (Figure 4A). An analysis of variance (ANOVA) with factors of condition (part-rearranged/control) and orientation (upright/inverted) revealed a significant interaction, *F*_(1, 53)_ = 4.76, *p* < 0.05. Specifically, participants reported perceiving the familiar region as figure more often for upright vs. inverted displays in the part-rearranged outline condition, *t*_(28)_ = 3.03, *p* < 0.05, but not in the control condition, *p* > 0.90.

Surprisingly, in the control condition, the familiar configuration was perceived as figure equally often in both upright and inverted displays, on 71% of trials for both orientations. These percentages are significantly above chance (50%), which suggests that object memories were influencing figure assignment, although no orientation dependency was observed. In what follows, we first attempt to understand why orientation-dependent OMEFA effects were not observed in the control prime condition. We then turn to a discussion of the orientation dependent effects obtained in the part-rearranged prime condition.

In order to understand the results obtained in the control condition, we return to considering our trial structure. In Experiment 1, the bipartite display was both post-masked and effectively pre-masked because a mask appeared after the outline silhouette prime that preceded the bipartite display (see Figure 3). The use of both pre- and post-masks effectively reduced the exposure of the bipartite test displays, which may have been a factor. It has been shown that it takes time for object memories to accumulate and strengthen (Jolicoeur, 1985; Corballis, 1988; Oram and Perrett, 1992), and in previous research, the orientation dependency of OMEFA effects was reduced in shorter compared to longer display exposures (Gibson and Peterson, 1994; Peterson and Gibson, 1994a). Another factor specific to the control condition is that the presentation of a novel outline silhouette comprising a novel collection of parts just prior to the bipartite display may have interfered with the build-up of the familiarity response to the critical region in the upright displays, which may also reduce the differential activity for upright and inverted bipartite displays. Perhaps participants in the control group based their figure responses on the one difference between the two regions that was discernible under these conditions—weak access to object memories in both upright and inverted orientations—producing above chance reports of the familiar region as figure but no orientation effects. There have been a few other reports of orientation-independent familiarity effects (Nelson and Palmer, 2007; Salvagio et al., 2011; Mojica et al., 2012). Participants in those experiments were detecting or discriminating targets briefly flashed on task-irrelevant bipartite displays. They responded faster when targets appeared on familiar regions than on the complementary regions, and this effect did not vary with display orientation. Mojica et al. showed that these effects were obtained only when the target location is highly variable producing uncertainty about where it might appear. Under these conditions, object properties available in the task-irrelevant displays implicitly guide participants' initial allocation of attention. In the service of fast detection/discrimination in those experiments, attention may be allocated before a differential familiarity signal has built up for upright vs. inverted displays^2^.

In contrast to the control group, participants in the part-rearranged prime group did show orientation-dependent OMEFA effects: they perceived the figure on the side of the border where a portion of a familiar object was sketched more often in upright than inverted displays. Thus, as predicted if the parts of a familiar object can access memories of the whole object even when they are spatially rearranged, the orientation-dependent OMEFA effects were larger for participants in the part-rearranged prime group than the control group. In the part-rearranged condition, as well as in the control condition, the trials were still effectively truncated by the pre- and post-masks. Moreover, for both groups, the prime configuration was novel, and its classification as novel likely interfered with the classification of the critical region of the bipartite display as familiar. The difference was that in the part-rearranged condition, but not the control condition, the parts of the prime silhouette were the same as those constituting the familiar object sketched on the familiar side of the upright bipartite test displays. That we obtained orientation-dependent OMEFA effects in this condition is consistent with the interpretation that, despite being rearranged, the upright parts in the part-rearranged primes activated representations of the whole upright familiar object that they typically constitute. This activation sums with the weak activation afforded by the bipartite test displays themselves, and results in greater activation of representations of familiar object for upright than inverted bipartite displays which in turn produces larger OMEFA effects for upright than inverted displays. The orientation-dependency of the familiar region as figure reports made by participants in the part-rearranged condition mark them as arising from access to the intact familiar configuration rather than simply its parts because familiar parts alone typically do not exert an orientation-dependent influence on figure assignment (Peterson et al., 1991; Gibson and Peterson, 1994).

We note that for upright displays, participants in the part-rearranged group did not perceive the familiar regions as figure more often than participants in the control group, whereas for inverted displays, they perceived the familiar regions as figure less often than participants in the control group. It is difficult to compare the absolute magnitudes of OMEFA effects in these two groups because familiarity was likely given different weights by the two groups. Our results show that orientation-dependent OMEFA effects emerge when object memories are differentially activated by upright and inverted displays.

## Experiment 2

In order for object memories to influence which region is perceived as figure, they must be accessed prior to the completion of figure assignment—that is, during the assessment of potential objects on opposite sides of a border and before one region has been assigned figural status. Previous studies have therefore taken these orientation-dependent OMEFA effects as evidence that object memory representations are accessed on a first, fast pass of processing through the visual system (Peterson et al., 1991, 2000; Peterson and Gibson, 1993, 1994a,b; Peterson, 1994; see Peterson and Cacciamani, 2013, for a review). Consistent with this view, more recent research shows that memories of object structure and coarse categorical information are accessed for objects suggested in regions that are ultimately perceived as shapeless grounds (Peterson and Skow, 2008; Peterson et al., 2012b; Sanguinetti et al., 2014; Cacciamani et al., unpublished manuscript). Because these regions were not assigned figural status, and therefore could not have accessed these representations after they were determined to be figures, these data stand as additional evidence that potential objects on both sides of borders are processed to high levels before one is determined to be the figure.

In Experiment 2, we asked whether the access to intact object memory representations by part-rearranged versions of those objects (as found in Experiment 1) can occur before figure assignment. To test this, we presented novel outline silhouettes before the bipartite test displays as in Experiment 1, but in Experiment 2, the rearranged parts of the familiar object were suggested on the groundside (outside) of the outline silhouette rather than on the figure side (inside; see Figure 2C). As in Experiment 1, the outline shapes were designed such that Gestalt configural cues biased the inside to be perceived as the figure; the outlines were small in area, symmetric about a vertical axis, enclosed, and surrounded. As such, we expected that the inside region would be perceived as the figure, and the outside would be perceived as a shapeless ground, and that participants would be unaware of the familiar parts suggested on the outside. Given the evidence showing that high-level object representations are accessed for grounds (Peterson and Skow, 2008; Peterson et al., 2012b; Sanguinetti et al., 2014; Cacciamani et al., unpublished manuscript), we investigated whether spatially rearranged collections of parts can access representations of the familiar objects they typically constitute before figure assignment. If they can, we should observe orientation-dependent OMEFA effects in Experiment 2, as we did in Experiment 1. Such a finding would replicate the effects observed in Experiment 1, and would show further that, even when arranged in a novel configuration, familiar parts access representations of familiar wholes before figure assignment has taken place.

### Materials and methods

#### Participants

A total of 16 undergraduate students from the University of Arizona participated in this experiment in order to partially fulfill course requirements. Prior to the experiment, all participants provided informed consent and reported normal or corrected-to-normal visual acuity.

#### Stimuli and apparatus

The bipartite test displays and the apparatus were the same as those used in Experiment 1. The outline silhouette primes that preceded the bipartite test displays differed from the part-rearranged primes in Experiment 1 in that in Experiment 2, the upright familiar parts were suggested along the outside—the ground side—rather than on the figure side (see Figure 2C). These primes were created from the part-rearranged primes in Experiment 1 by flipping the left and right borders about their vertical axes. Doing so placed the rearranged familiar parts along the ground side of the prime. All other aspects pertaining to the prime—including its visual angle, location, and exposure duration—were the same in Experiment 2 as they were in Experiment 1.

#### Design and procedure

The design and procedure were the same as in Experiment 1. In post-experiment questioning, participants were again shown an example outline silhouette (that had not been used during the experiment) while the experimenter pointed out and traced the familiar, rearranged parts suggested in the ground. They were then directly asked if, during the experiment, they noticed any familiar parts on the groundside of the prime and whether the prime influenced their figure decision. None of the participants reported having perceived the familiar, rearranged parts suggested along the groundside of the outlines or their relationship to the bipartite test display in Experiment 2. Given these reports and the Gestalt configural cues biasing the inside of the outline shape to be perceived as the figure, we are reasonably confident that participants perceived the inside as figure and were not aware of the rearranged parts suggested in the seemingly shapeless ground, nor of the relationship between the silhouettes and the familiar region in the bipartite test display.

### Results and discussion

The results of Experiment 2 can be seen in Figure 4C. When the familiar, rearranged parts were suggested on the groundside—rather than the figure side—of the outline shape that preceded the bipartite test display, orientation-dependent OMEFA effects were again observed. Participants reported seeing the familiar region as the figure significantly more often when it was upright vs. inverted, *t*_(25)_ = 2.85, *p* < 0.05. An ANOVA comparing responses made by participants in Experiment 2 to those of participants in the control group from Experiment 1 revealed a significant interaction between group and orientation, *F*_(1, 40)_ = 4.34, *p* < 0.05.

Experiment 1 and 2 together showed that familiar but spatially rearranged parts are sufficient to access representations of the whole familiar configuration. This effect was found regardless of whether the parts were suggested on the figure side or ground side of the outline; a 2 × 2 ANOVA with factors of experimental condition (rearranged parts on figure vs. ground) and orientation of the test display (upright vs. inverted) revealed no significant differences in figure responses between the part-rearranged conditions in Experiments 1 and 2 (*p*s > 0.50). This suggests that even when spatially rearranged, parts can implicitly facilitate perception of the intact whole with the same collection of familiar parts in a different spatial relationship, and that this occurs before figure assignment.

## General discussion

This study investigated whether prior presentation of object parts can implicitly influence perception of the whole familiar object, even when those parts are arranged in a novel configuration. We found that a novel outline of a silhouette containing the spatially rearranged parts of an upcoming familiar configuration facilitated orientation-dependent OMEFA effects. That is, the presence of an object's parts, though spatially rearranged into a novel configuration, made participants more likely to report a region depicting an intact version of that object as the figure when it was suggested in its upright vs. inverted orientation in a test display. This finding indicates that the familiar parts of an object, even when spatially rearranged into a novel configuration, activate the representation of the whole familiar object that they typically constitute. This orientation-dependent effect emerged when compared to a control condition in which the parts of the novel outline silhouette had no relationship to the upcoming test display. In this control condition, figure reports did not differ for upright vs. inverted displays, though in both orientations, the familiar region was reported as figure more often than chance.

Moreover, Experiment 2 revealed that this activation of the whole object via presentation of its parts occurs prior to figure assignment. Specifically, even when the rearranged parts were suggested on the side of the outline's borders ultimately perceived as a shapeless ground, they still activated a representation of the whole configuration such that orientation-dependent OMEFA effects were obtained. Therefore, the results of Experiment 1 and 2 together show that both the whole as well as the parts that comprise it are taken into account early in the course of object perception.

This is the first study showing that, compared to a control condition, orientation-dependent OMEFA effects are facilitated via prior presentation of the spatially rearranged parts of the upcoming familiar configuration. One other study showed that OMEFA effects were facilitated by the prior presentation of the whole familiar configuration compared to a no-prime condition (Gibson and Peterson, 1994). The results of the present study extend those results by showing that facilitation can also be observed via priming with spatially rearranged parts. However, it should be noted that whereas Gibson and Peterson compared figure reports on upright displays only, our measure of interest is the difference in reports for upright vs. inverted familiar configurations. Additionally, Gibson and Peterson's primes were exposed for long durations (2 s) and were not masked (though their bipartite displays were).

Although some research shows that parts and wholes are represented separately in the brain (Baker et al., 2002; Davidoff and Roberson, 2002; Behrmann et al., 2006; Yamane et al., 2006), the current study supports recent work showing that these representations can interact (Barense et al., 2012; Peterson et al., 2012a). In their fMRI experiment, Peterson et al. found that in addition to responding to the familiarity vs. novelty of configurations, the PRC of the MTL assesses the familiarity of collections of parts, even when they are spatially rearranged so as to constitute a novel configuration. This finding raised the possibility that collections of familiar parts might activate memory representations of the intact whole object they typically constitute. We have provided evidence that this occurs by showing that part-rearranged configurations can facilitate perception of an upcoming familiar object comprising those parts.

The present study also concurs with previous work on parts and wholes in the area of face recognition. Although much of the face perception literature has shown that the whole configuration of a face dominates our perception over the individual parts (e.g., Tanaka and Farah, 1993; Barton et al., 2002), recent work suggests that the parts of a face are still represented in cortical regions traditionally associated with face-specific processing, such as the fusiform gyrus and the occipital face area (Yovel and Kanwisher, 2004; Pitcher et al., 2007; Harris and Aguirre, 2008, 2010; Liu et al., 2010). Moreover, this face part processing occurs early in time, as ascertained via magnetoencephalography, which has high temporal resolution (Harris and Aguirre, 2008). Our results are consistent with those observed in the face perception literature, as we have found that although parts of a familiar object that are spatially rearranged to form a novel configuration are not recognized as familiar, they nevertheless activate the representation of the intact whole object. Additionally, we have shown that access to the intact whole object occurs early in time before figure assignment has occurred.

### Open questions

When considered in the context of previous research, the current study raises a number of questions that will be addressed, where possible, by future research. We discuss those questions next in the context of the relevant previous research.

First, Peterson et al.'s (2012a) neuroimaging work (see also Barense et al., 2012) suggests that when the PRC detects a mismatch in the familiarity of a collection of parts and the configuration they constitute (as in a part-rearranged novel configuration), it suppresses familiarity signals in lower visual areas that represent part familiarity. In the present study, we have no way of ascertaining whether the familiarity responses to the parts of the part-rearranged outline prime were suppressed. Perhaps low-level part familiarity responses were indeed suppressed when participants viewed the part-rearranged prime, but suppression of part familiarity responses did not prevent us from observing priming resulting from the access to the intact familiar configuration. The suppression of the familiarity response to the parts of the prime may have even reduced the observed magnitude of the orientation-dependent OMEFA effects, although we have no way of knowing for certain. That we obtained our orientation-dependent OMEFA effects despite this possibility speaks to the robustness of those effects.

Second, inhibitory models of object perception posit that when two regions share a border (as in our bipartite test displays), they engage in inhibitory competition for figural status (e.g., Kienker et al., 1986; Grossberg, 1994; Roelfsema et al., 2002; Jehee et al., 2007). The winner of this competition is perceived as the figure, while the loser is seen as a shapeless ground, and moreover, is suppressed (Likova and Tyler, 2008; Peterson and Skow, 2008; Salvagio et al., 2012). If grounds are suppressed, then one might ask why we did not observe reduced reports of the familiar region as figure on test displays following outlines whose parts were present on the ground vs. figure side of the border (i.e., reports in the part-rearranged condition Experiment 2 vs. 1, respectively, which were in fact equivalent). One explanation is based on the finding that the amount of suppression applied to the ground depends on the degree to which it engages in competition for figural status (Salvagio et al., 2012), with greater competition leading to greater suppression (cf., Peterson, 2012). In our outline displays in Experiment 2, even though they are composed of familiar parts, the grounds suggest novel configurations. Because of this, the engagement of the ground region in competition for figural status is low, especially given all the cues favoring the inside region as the figure (i.e., small area, surroundedness, enclosure, and symmetry). Given this low amount of competition from the ground, the suppression applied there likely was also low, which may have been why we did not observe effects of ground suppression in behavioral responses in Experiment 2 where the parts were present in the ground. Additionally, previous work has indicated that ground suppression is short-lived; effects of ground suppression on behavioral responses are only evident at prime-to-target stimulus onset asynchronies (SOAs) of 100 ms or shorter (Peterson and Skow, 2008; Salvagio et al., 2012). In the present study, the test display appeared 270 ms after the onset of the outline silhouette prime, which may have been too long for ground suppression to exert a measurable influence on behavior. Consequently, no difference in figure reports was observed as a function of whether the rearranged parts appeared on the figure or ground side of the outline's borders.

The above discussion on figure vs. ground raises an important point. We assume in this study that participants perceived the inside of the outline prime as the figure. This assumption is critical to our interpretation of Experiment 2 where the parts were suggested on the outside (ground side) of the outline's borders. However, we are reasonably confident that participants did indeed perceive the inside as figure and the outside as the ground based on the presence of Gestalt configural cues—including small area, closure, surroundedness, and symmetry—which favored this percept. Additionally, the outlines were always presented at fixation and were expected, which also increases the interpretation of the central, inside region as the figure (Peterson and Gibson, 1994b; Vecera et al., 2004). Importantly, regardless of the percept, participants reported not perceiving the familiar parts that comprised the outline (except for three participants in Experiment 1, who were eliminated from the analysis). Thus, we can also be reasonably confident that the effects observed in these experiments are implicit.

Finally, we interpreted our difference between figure reports for upright vs. inverted displays in the part-rearranged condition as facilitation of orientation-dependent OMEFA effects. However, an alternative explanation could be that the upright vs. inverted difference arose due to interference from the rearranged parts in the prime on the processing of the inverted displays. Upon the appearance of the part-rearranged prime, the representations of those upright parts would be activated such that the visual system was primed for upright stimuli. In this way, it would be more difficult for the system to then process and activate representations for an inverted stimulus. This interference in the inverted condition might have led to decreased reports of the familiar configuration as figure as compared to the upright condition. This interpretation of our data would still support our claim that prior presentation of object parts can implicitly activate the representation of the whole, although the influence on perception would be in the form of interference rather than facilitation. Future studies should investigate this alternative explanation.

## Summary

In two experiments, we show that orientation-dependent object memory effects on figure assignment can be facilitated via prior presentation of the rearranged parts of the upcoming familiar configuration compared to a control condition. These results are consistent with the interpretation that familiar parts can access representations of familiar wholes even when they are spatially rearranged into a novel configuration, and therefore have enormous implications for understanding the mechanisms underlying object perception.

### Conflict of interest statement

The authors declare that the research was conducted in the absence of any commercial or financial relationships that could be construed as a potential conflict of interest.

## Footnotes

^1^Prior to this experiment, all upright bipartite stimuli were piloted to ensure agreement across observers on the identity of the familiar configuration. In order to be included in the experiment, a stimulus had to have at least 65% agreement (less than 35% disagreement) across observers on the interpretation of the familiar region. These common interpretations are the ones listed in Supplementary Material. In this way, we are reasonably confident that our stimuli are ones about which most participants are able to consciously access object memories.

^2^ Cf. Shomstein (2012), for an explanation of object-based attention effects as a strategy employed under conditions of uncertainty.
