# Supplementary material for: Spatially rearranged object parts can facilitate perception of intact whole objects
Source: Front Psychol. 2014 May 27;5:482. doi: 10.3389/fpsyg.2014.00482 (PMC4033907; doi:10.3389/fpsyg.2014.00482)
Supplement: Supplementary file 1 [file DataSheet1.DOCX]

**Appendix A**

List of familiar configurations depicted in the familiar regions of the bipartite test displays.

| Anchor | Hydrant |
| --- | --- |
| Apple | Jet |
| Bear | Lamp |
| Bell | Leaf |
| Bulb | Pear |
| Bunny | Pineapple |
| Coffee pot | Seahorse |
| Dog | Windmill |
| Duck | Toilet |
| Eagle | Train |
| Face | Tree |
| Flower | Trumpet |
| Grapes | Umbrella |
| Guitar | Wine glass |
| Hand | Woman |
| House | Wrench |
